# The influence of biological DMARDs on aseptic arthroplasty loosening: a retrospective cohort study

**DOI:** 10.1093/rheumatology/kead304

**Published:** 2023-07-04

**Authors:** Markus M Schreiner, Jennifer Straub, Sebastian Apprich, Kevin Staats, Reinhard Windhager, Daniel Aletaha, Christoph Böhler

**Affiliations:** Department of Orthopedics and Trauma Surgery, Medical University of Vienna, Vienna, Austria; Department of Orthopedics and Trauma Surgery, Medical University of Vienna, Vienna, Austria; Department of Orthopedics and Trauma Surgery, Medical University of Vienna, Vienna, Austria; Department of Orthopedics and Trauma Surgery, Medical University of Vienna, Vienna, Austria; Department of Orthopedics and Trauma Surgery, Medical University of Vienna, Vienna, Austria; Department of Rheumatology, Medical University of Vienna, Vienna, Austria; Department of Orthopedics and Trauma Surgery, Medical University of Vienna, Vienna, Austria

**Keywords:** aseptic loosening, biological DMARDs, total hip arthroplasty, total knee arthroplasty, RA

## Abstract

**Objective:**

To investigate whether biological DMARDs affect the risk of aseptic loosening after total hip/knee arthroplasty (THA/TKA) in patients with RA.

**Methods:**

We retrospectively identified all patients suffering from RA who underwent THA/TKA at our academic centre between 2002 and 2015 and linked them with an existing prospective observational RA database at our institution. The risk of aseptic loosening was estimated using radiological signs of component loosening (RCL). A time-dependent Cox regression analysis was used to compare the risk of implant loosening between patients treated with traditional DMARDS and biological DMARDs, or alternately both over time.

**Results:**

A total of 155 consecutive total joint arthroplasties (TJAs) (103 TKA *vs* 52 THA) was retrospectively included in the study. Mean age at implantation was 59 ± 13 years. Mean follow-up time was 69 ± 43 months. Overall, 48 (31%) TJAs showed signs of RCL, with 28 (27.2%) RCLs occurring after TKA compared with 20 after THA (38.5%). A significant difference regarding the incidence of RCL between the traditional DMARDs group (39 cases of RCL, 35%) and the biological DMARDs group (nine cases of RCL, 21%) (*P* = 0.026) was observed using the log-rank test. This was also true when using a time-dependent Cox regression with therapy as well as arthroplasty location (hip *vs* knee) as variables (*P* = 0.0447).

**Conclusion:**

Biological DMARDs may reduce the incidence of aseptic loosening after TJA in patients with RA compared with traditional DMARDs. This effect seems to be more pronounced after TKA than THA.

Rheumatology key messages

- Biological DMARDs are associated with a significantly lower incidence of RCL after TJA in patients with RA.
- Biological DMARDs affect the rate of RCLs more pronounced in patients after TKA than THA.

## Introduction

RA is a chronic inflammatory disease that, if treated too late or inadequately, can lead to permanent joint destruction [1]. Recent advances in treatment, including early and aggressive use of DMARDs [2, 3] as well as a combination of traditional DMARDs and biological DMARDs, have resulted in significant reductions in morbidity and incidence of end-stage joint damage in RA patients [4, 5]. Despite these improvements, the number of RA patients requiring total joint replacement of a large joint remains sizable [6]. While the cost effectiveness of TJA, its ability to reduce pain and improve function are well documented [7, 8], it may be associated with serious complications such as aseptic loosening, periprosthetic joint infections (PJI), dislocation and periprosthetic fractures [9–11]. Compared with patients undergoing TJA for OA, RA patients have a higher risk for revision surgery due to PJI after both TKA [12, 13] and THA [13] as well as a higher risk of dislocation after THA [12]. Despite these differences, aseptic loosening is the most common cause of revision TJA in RA patients and OA patients after THA [10, 11] and, depending on the literature, the most common [9, 14] or second most common cause of revision TJA after TKA [15]. An analysis of worldwide arthroplasty registries found aseptic loosening to be responsible for 55% of revisions after THA compared with 29.8% after TKA. Overall, in this study, one out of 13 patients after THA had to be revised at some point due to aseptic loosening [16]. In a study based on data of the Australian joint registry, the rate of aseptic loosening after THA in RA patients averaged 14.3% at 15 years [17]. Revision surgery for implant failure due to aseptic loosening is associated with loss of bone stock, decreased clinical outcome and higher risk of subsequent complications. Therefore, a medical intervention that prevents or lowers the risk of aseptic loosening would be of utmost importance [18].

The reasons for aseptic loosening have not yet been fully elucidated, but local inflammation due to prosthetic wear debris, its phagocytosis by macrophages and the subsequent secretion of bone resorption inducing cytokines, most notably TNF, is thought to play a key role [19]. Hence, TNF inhibitors may be promising therapeutic candidates to reduce the incidence of aseptic loosening. This theory is corroborated by the fact that different studies have shown that TNF inhibitors can prevent the formation of erosions in RA [4, 20, 21]. Furthermore, a recent study that investigated the influence of disease activity on rates of RCL found that treatment with biological DMARDs was associated with lower rates of RCL when compared with treatment with traditional DMARDs [22]. However, larger studies are needed to confirm this theory.

Hence, the aim of this study was to investigate whether biological DMARDs affect the risk of aseptic loosening after total hip/knee arthroplasty in patients with RA compared with conventional DMARDs.

## Materials and methods

This retrospective single-centre study was conducted in accordance with the Declaration of Helsinki, and the ethics committee of the Medical University of Vienna has approved this study.

### Patient cohort

We retrospectively identified all patients who underwent THA/TKA at our institution between 2002 and 2015 and linked them with an existing prospective observational RA database at our institution. All participants fulfilled the 1987 ACR [23], or more recently, the 2010 ACR/EULAR classification criteria for RA [24]. Based on their antirheumatic medication, patients were divided into two groups: patients under therapy with traditional DMARDs and patients under therapy with biological DMRADs ± traditional DMARDs. RA patients are closely monitored at the Department of Rheumatology with outpatient visits every three to four months. Patients who did not have continuous documentation of their RA treatment during the study period or patients who were lost to follow-up before the two-year follow-up had to be excluded. Demographic data of the patient cohort were retrospectively identified. Furthermore, all patients who underwent revision surgery were identified and differentiated between revision for aseptic loosening, periprosthetic joint infection and soft-tissue complications.

### Radiological analysis

Because revision surgery for aseptic loosening was not frequently enough observed in our cohort to be used as a primary end point, radiological signs for aseptic component loosening (RCL) were employed as a more sensitive surrogate parameter instead. Even though the presence of RCL does not necessarily indicate a loose implant, it is directly correlated with aseptic loosening and may predict revision surgery for aseptic loosening [25, 26]. Routine radiological follow-up at the Department of Orthopaedics and Trauma Surgery was performed after 6 weeks, 3 months, 12 months and then yearly. For patients after THA, the acetabular component was assessed on anterioposterior radiographs according to the zones described by DeLee and Charnley [27], the femoral stem was assessed on anterioposterior and lateral X-rays according to the Gruen zones [28]. For patients after TKA, the Knee Society Roentgenographic Evaluation System [29] was used to assess anterioposterior and lateral X-rays of the knee joint. RCL was defined if one of the following were true in at least one region around the implant: radiolucent lines (RLL) >2 mm; osteolysis defined as nonlinear areas of endosteal, intracortical, or cancellous bone destruction >2 mm or migration of implant components of >2 mm [30]. All radiographic evaluations were performed on a picture archiving and communication system (PACS) workstation (IMPAX EE R20, Agfa Healthcare N.V., Mortsel, Belgium) by an expert reader with more than eight years of experience in the evaluation of radiological studies. Imaging studies were assessed in random order and the reader was blinded to all patient details.

### Statistical analysis

Metric data are described using mean ± standard deviation. To assess the risk of RCL according to medication, Kaplan–Meier survival analysis was performed with medication type as independent factor and RCL as end point. Patients were censored if they reached their last observation. Log-rank testing was used to compare groups for significant differences. Whereas for most patients the antirheumatic treatment remained constant during the entire follow-up period on either traditional DMARDs or biologicals DMARDs ± traditional DMARDs, some patients switched between both treatment regimes. Because Kaplan–Meier analysis cannot adequately account for patients changing therapy, we performed a total of three Kaplan–Meier analyses in order to take a comprehensive and unbiased statistical approach, assigning the patients who changed therapy during follow-up to the respective groups according to different rules in each case. For the first analysis, those patients who received both treatment regimens during follow-up were assigned to the treatment they had received longer. For the second analysis, patients who had received a biological DMARD at least once during follow-up were allocated to the biological DMARD group. For the third analysis all patients who had received a traditional DMARD exclusively at least for one month during follow-up were allocated to the traditional DMARD group. To be able to account for these therapy switches, we further conducted a time-dependent Cox regression analysis to compare the risk of implant loosening between patients treated with traditional DMARDs and biological DMARDs, or alternately, both over time. Implant location was additionally included into our model. The proportional hazards assumption was confirmed by visual assessment of the Kaplan–Meier curves, and the final model was tested for significance using likelihood ratio testing.

To assess any difference in treatment quality over time during the long secular period between 2002 and 2015, disease activity using the Simplified Disease Activity Index (SDAI) was retrospectively identified and employed as a surrogate parameter. Time-integrated level of disease activity (area under the SDAI curve) was calculated and compared using a scatter plot and regression analysis to determine potential differences in SDAI_AUC_ based on the date of implantation of the TJR.

Dichotomous variables were compared via χ^2^ testing or Fisher's exact test in case of less than five observations. Continuous variables were assessed via *t* tests or Wilcoxon signed-rank tests depending on their distribution.

All calculations were performed in their two-sided versions at a significance level of 0.05 using SPSS v28.0 (IBM SPSS Statistics for Windows, v28.0. Armonk, NY, USA), except for the time-dependent Cox regression calculations, which were done in R (v4.1.3) [31].

## Results

### Study population

We identified 155 consecutive TJAs in 96 patients (75 female, 21 male), who met the inclusion criteria and were retrospectively enrolled in the study. TKA was performed in 103 cases and THA in 52 cases. Mean age at implantation was 59 ± 13 years. Mean follow-up time was 62 ± 42 months. Age at implantation differed significantly between cases who were treated with traditional DMARDs (61 ± 12 years) *vs* biological DMARDs (55 ± 2 years) (*P* = 0.02). The follow-up time was significantly longer for cases who were treated with biological DMARDS (74 ± 7 months) when compared with cases treated with traditional DMARDs (58 ± 39 months) (*P* = 0.03) (Table 1).

**Table 1.** Numbers indicate median and standard deviations except where indicated otherwise

	Cases with predominantly traditional DMARDs (*n* = 113)	Cases with predominantly biological DMARDs (*n* = 42)	*P*-values
Age at surgery, years	61 ± 12	55 ± 2	*P* = 0.0213
Gender	F = 83, M = 30	F = 37, M = 5	*P* = 0.0526
Location			
TKA	82	21	*P* = 0.0237
THA	31	21	
FU-time, months	58 ± 39	74 ± 7	*P* = 0.0279
Charlson Comorbidity Index	3.0 ± 1.7189	3.0 ± 1.7088	*P* = 0.2167
Type of treatment at implantation	Biological DMARDs: 3	Biological DMARDs: 28	NA
	Traditional DMARDs: 110	Traditional DMARDs: 14	
Overall revisions	13 (11.5%)	2 (4.7%)	*P* = 0.2069
Revision for aseptic loosening	7 (6.2%)	2 (4.7%)	*P* = 0.7346

NA, not applicable.

A total of 102 TJAs were exclusively treated with traditional DMARDs during the entire follow-up, whereas for 33 TJAs the antirheumatic treatment included biological DMARDS during the entire follow-up. In contrast, 20 TJAs switched between being treated using traditional DMARDs exclusively and biological DMARDs ± traditional DMARDs at some point during their follow-up.

### Disease activity

**Figure 1.**
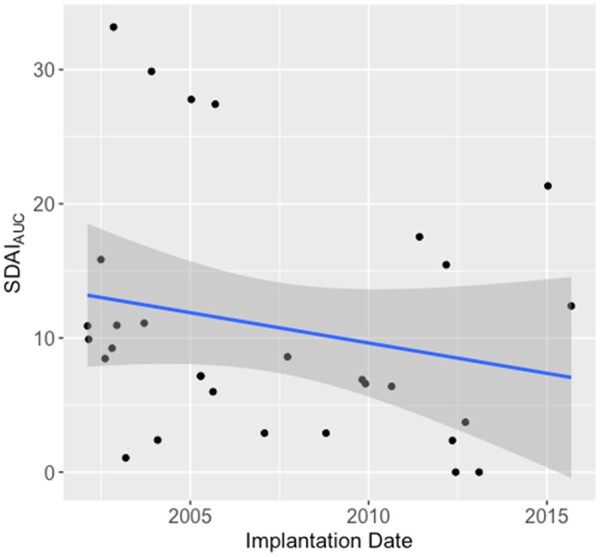
Scatter plot depicting the time-integrated level of disease activity using the area under the Simplified Disease Activity Index curve (SDAI_AUC_) according to the date of implantation of the total joint replacement of the subset of patients with continuous documentation of their SDAI scores (*n* = 29)

**Figure 2.**
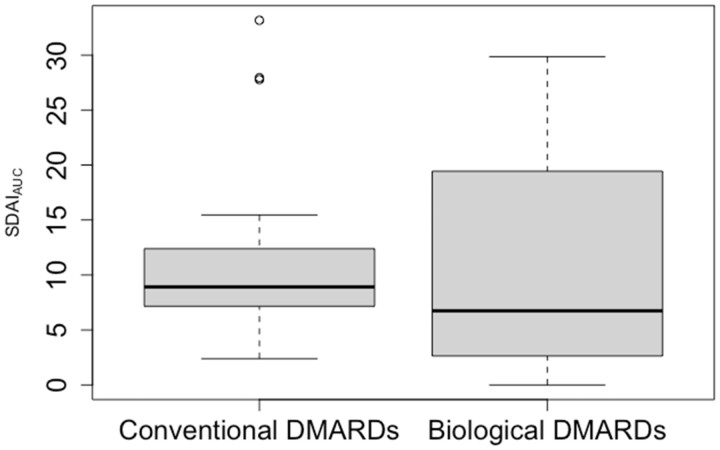
Boxplot comparing the time-integrated level of disease activity using the area under the Simplified Disease Activity Index curve (SDAI_AUC_) in the subset of patients with continuous documentation of their SDAI scores (*n* = 29) between patients who were treated predominantly with biological DMARDs (*n* = 14) (10.94 ± 10.20 SDAI_AUC_) *vs.* patients who were treated predominantly with conventional DMARDs (*n* = 15) (11.90 ± 8.78 SDAI_AUC_)

SDAI scores were available for a subset of 29 patients, 14 of which were treated with biological DMARDs and 15 with conventional DMARDs. In this subset of patients, we did not observe a strong trend regarding disease activity across the observed time frame of inclusion (Fig. 1). Furthermore, we found similar time-integrated SDAI scores between patients, who were predominantly treated with biological DMARDs (10.94 ± 10.20 SDAI_AUC_) *vs.* patients who were predominantly treated using conventional DMARDs (11.90 ± 8.78 SDAI_AUC_, Fig. 2).

### Revision surgeries

A total of 16 revision surgeries were observed during follow-up. One revision was due to a septic complication, one for a periprosthetic fracture, one for a liner exchange, three after soft tissue complications and one for secondary patellar resurfacing. Aseptic loosening was the most common reason for revision with nine observed revision surgeries during the follow-up period, seven of which occurred in patients treated with traditional DMARDs *vs.* two treated with biological DMARDs (n.s.).

### Impact of antirheumatic therapy on radiological signs for aseptic component loosening

**Figure 3.**
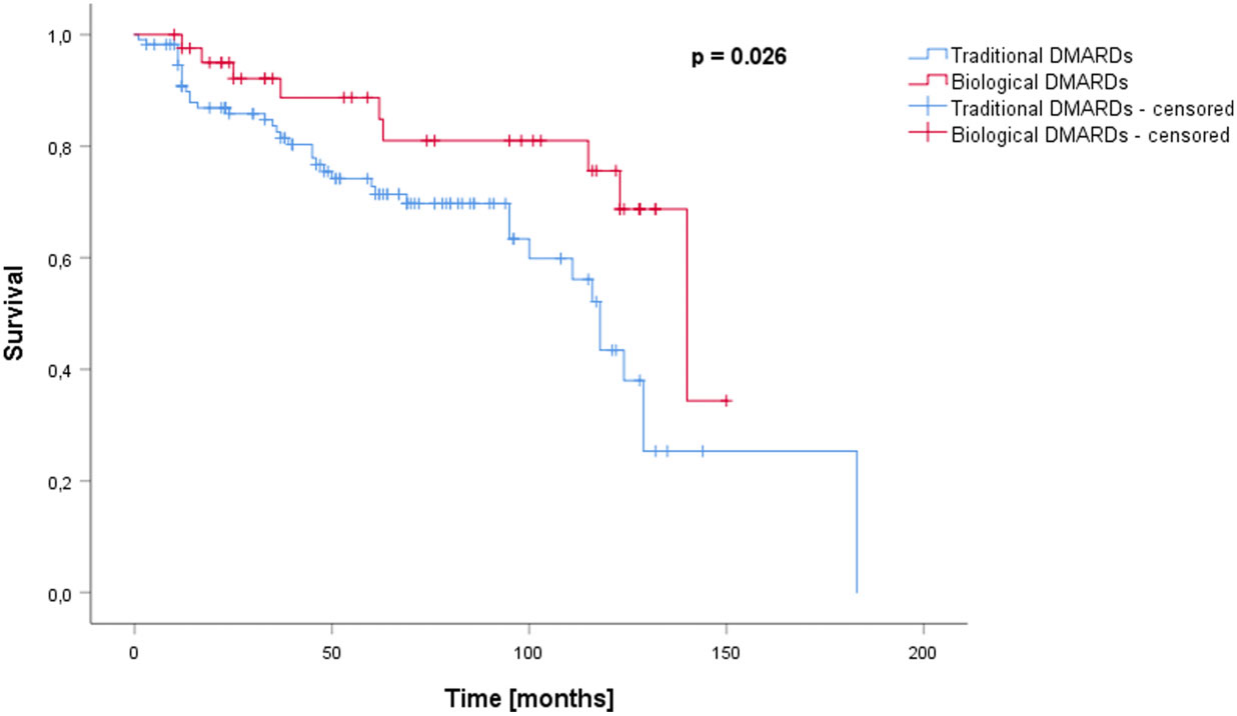
Survival (follow-up time until lost-to-follow-up or RCL) in months for patients treated with traditional DMARDs *vs.* patients treated with biological DMARDs. For this analysis, patients who switched treatment regimens during follow-up (*n* = 20) were allocated to the treatment they received longer. Survival differed significantly (log-rank test *P* = 0.026)

Overall, 48 (31%) patients showed signs of RCL with 28 (27.2%) radiolucencies occurring after TKA compared with 20 after THA (38.5%). When assigning the patients, who switched between treatment regimens to the therapy they had received longer, a significant difference was observed in the Kaplan–Meier analysis and log-rank test regarding the incidence of RCL between the traditional DMARDs group (39 cases of RCL, 35%) and the biological DMARDs group (nine cases of RCL, 21%). (Fig. 3) (*P* = 0.026).

**Figure 4.**
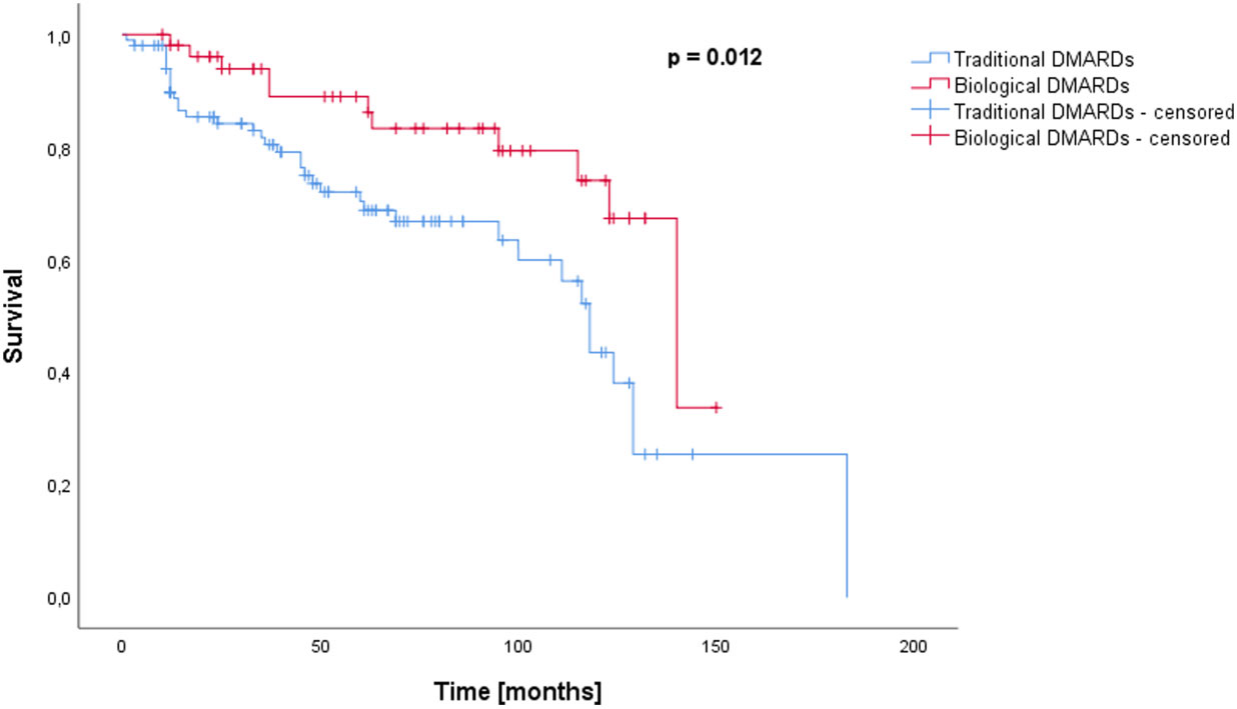
Survival (follow-up time until lost-to-follow-up or RCL) in months for patients treated with traditional DMARDs *vs.* patients treated with biological DMARDs. For this analysis, all patients who received a biological DMARD at least once during follow-up were allocated to the biological DMARDs group. Survival differed significantly (log-rank test *P* = 0.012)

When assigning patients who had received a biological DMARD at least once during follow-up to the biological DMARD group, a significant difference was observed in the Kaplan–Meier analysis and log-rank test regarding the incidence of RCL between the traditional DMARDs group and the biological DMARDs group as well (*P* = 0.012) (Fig. 4).

**Figure 5.**
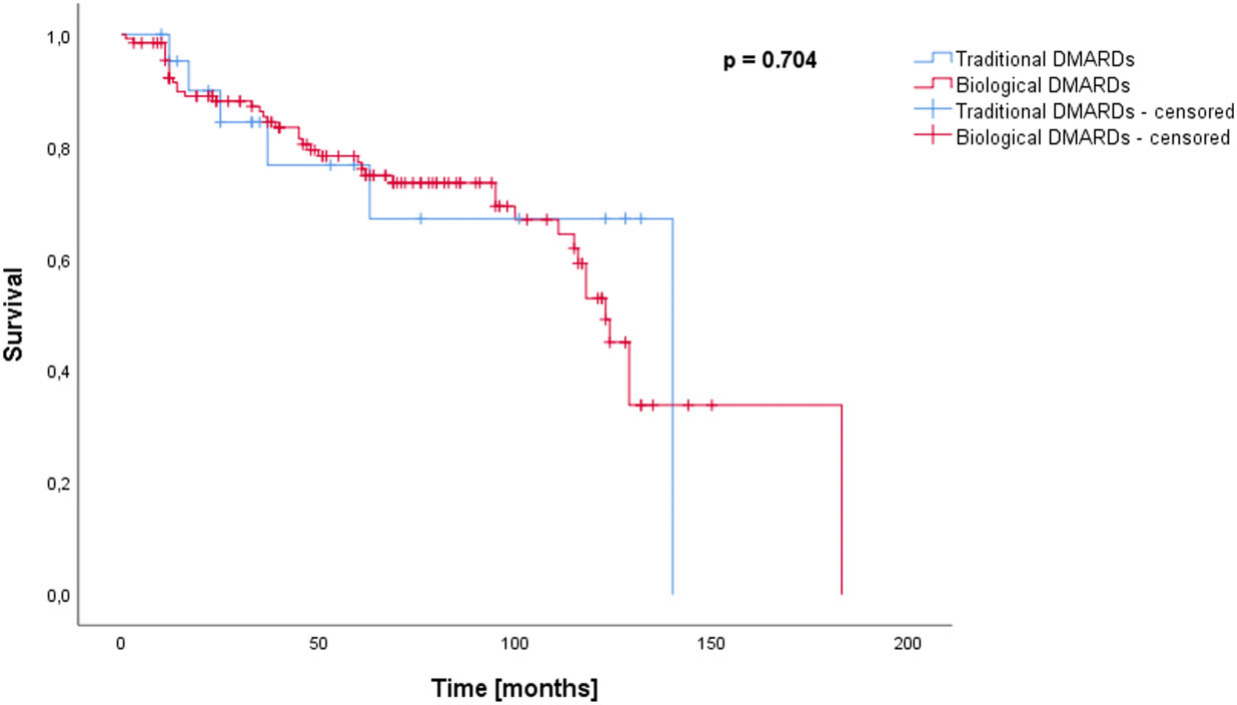
Survival (follow-up time until lost-to-follow-up or RCL) in months for patients treated with traditional DMARDs *vs.* patients treated with biological DMARDs. For this analysis, all patients who received a treatment with traditional DMARD only at least once during follow-up were allocated to the traditional DMARDs group. Survival did not differ significantly (*P* = 0.704)

When assigning patients who had received a traditional DMARD exclusively at least for one month during follow-up to the traditional DMARD group, no significant difference was observed in the Kaplan–Meier analysis and log-rank test regarding the incidence of RCL (*P* = 0.704) (Fig. 5).

Using a time-dependent Cox regression with therapy (odds ratio: 0.4597; 95% confidence interval [0.2180, 0.9687]) as well as arthroplasty location (hip *vs* knee) (odds ratio: 1.7961; 95% confidence interval [0.9851, 3.2744]) as variables demonstrated a significant difference regarding the occurrence of RCL between treatment with traditional DMARDs *vs.* biological DMARDS (*P* = 0.0447) as well.

This difference in RCL rates between traditional DMARDs and biological DMARDs was more pronounced in the TKA group with 25 of 82 (30.5%) patients under traditional DMARDS compared with 3 of 21 (14.3%) of patients under biological DMARDs showing signs of RCL. In the THA group, 14 of 31 (45.2%) patients under traditional DMARDs and 6 of 21 (28.6%) patients under biological DMARDs showed signs of RCL.

## Discussion

This is the first study to investigate the impact of biological DMARDs on aseptic arthroplasty loosening after total hip and knee replacement in RA patients and demonstrates a reduced incidence of RCL in patients treated with biological DMARDs when compared with therapy with traditional DMARDs.

Despite advances in material science that reduced implant-wear significantly, aseptic loosening remains the primary failure mode after total joint arthroplasty for RA as well as OA [32, 33]. Because the number of total joint replacements (TJR) will most likely continue to increase in the coming years [7–9], aseptic loosening as well as revision TKA and revision THA are set to continue to increase as well [34]. Revision for aseptic loosening has grave implications and is associated with high morbidity, bone loss, increased risk of complications and worse clinical outcome. So far, no medical intervention to avoid or reduce the risk of aseptic loosening has been identified.

The exact mechanism behind aseptic loosening remains unknown; however, local inflammation due to prosthetic wear is thought to be a key driver. The evaluation of periprosthetic membranes, which had been retrieved from the bone-cement interface of loose hip prostheses, demonstrated an association between bone resorption and the presence of small enough wear particles (diameter 1–12 µm) to allow them to be phagocytosed but not digested by macrophages [35]. Macrophages, when exposed to wear particles of this size *in vitro*, have been observed to phagocytose these wear particles and secrete TNF, which in turn may lead to the observed bone resorption and prosthetic loosening [36]. Other bone resorption promoting cytokines, which are released due to the unsuccessful digestion of phagocytosed wear particles, include IL-1, IL-5, IL-17 as well as M-CSF [37]. In addition, it has been shown that already the binding of wear debris to the cell surface of phagocytes is sufficient to induce the secretion of IL-1 and TNF [19]. Merkel *et al.* subsequently successfully tested the hypothesis that TNF mediates implant osteolysis in a murine model. The authors observed that mice deleted of both the p55 and p75 TNF receptors are not susceptible to the bone resorption caused by polymethyl-methacylate (PMMA) particle implantation [38].

Interestingly, it has been observed that in aseptic loosening the resorbed bone was replaced with a synovial-like membrane [39]. While these membranes differed from those found in RA in terms of histopathology and triggering mechanisms, they resemble the pannus of RA in terms of its tendency to produce localized cytokine-mediated bone loss [18]. Fittingly, a recent study found that higher inflammatory disease activity in RA patients increases the risk for radiographic signs of component loosening (RCL) [22], making the argument that systemic inflammation in RA might influence this process of local inflammatory-mediated osteolysis.

There are certain limitations to this study that have to be addressed. First and foremost, this study was conducted using a retrospective study design. However, due to the lack of supporting data, this was the logical first step to investigate our hypothesis. Furthermore, despite the retrospective study design, the study population is relatively small. However, it has to be considered that the availability of a comprehensive orthopaedic and radiological follow-up in combination with a gapless documentation of the antirheumatic therapy is rare. In addition, our study’s long secular period may introduce bias due to differences in patient characteristics and treatments between 2002 and 2015. However, when assessing Simple Disease Activity Index (SDAI) scores, which were available for a subset of 29 patients, only minor numerical differences were found over the inclusion window. Unfortunately, being only available for 29 patients, a stratification for disease activity was not feasible, which is another limitation of this study. However, when comparing the SDAI_AUC_ in the subset of patients with continuous disease activity monitoring, we found similar disease activity between patients who were predominantly treated with conventional DMARDs *vs* patients who were predominantly treated with biological DMARDs (Fig. 2). Furthermore, the data were collected from a tertiary referral centre. Hence, a certain selection bias cannot be ruled out. However, our centre treats a mixed population of primary, secondary and tertiary referrals, covering both simple and challenging cases of RA. Hence, we consider this group to be representative of the full spectrum of RA patients and disease courses. Even though revision surgery of aseptic loosening was observed more frequently in patients treated with traditional DMARDs in comparison with patients treated with biological DMARDs, the number of patients was insufficient to use revision surgery for aseptic loosening as a primary end point. Instead, radiological signs of aseptic loosening were chosen. Whereas RCLs do not warrant immediate intervention in the clinical orthopaedic setting and don’t necessarily indicate a loose implant, their presence is closely correlated with revision surgery for aseptic loosening and might even predict it. In addition, it could be argued that the decision to do revision surgery for aseptic loosening is somewhat subjective and other characteristics of the patient such as comorbidities might also be taken into account.

Furthermore, there was a significant difference in age at implantation between patients treated with biological DMARDs *vs* traditional DMARDs. However, younger age at implantation is commonly associated with higher demand and higher physical activity and should cause the opposite of what was observed: a higher rate of RCL in the biological DMARDs group. Likewise, the follow-up time was significantly longer for patients treated with biologicals when compared with patients with traditional DMARDs. This, however, would also lead to a higher rate of RCL in the biological DMARDs group. Lastly, we cannot rule out bias by indication regarding the decision of which patients were treated with biological DMARDS *vs* traditional DMARDs. However, it can be assumed that patients with higher disease activity are more likely to be treated using a biological DMARD, which would further substantiate our findings.

We conclude that treatment with biological DMARDs seems to reduce the risk of RCL in RA patients undergoing TJA. This risk reduction seems to be more pronounced after TKA than after THA. Whether this effect of biological DMARDs on the risks of RCL could also be observed in patients undergoing TJA for OA can only be hypothesized. Additional prospective studies are needed to further investigate these first findings.

## Data Availability

The data underlying this article cannot be shared publicly for the privacy of individuals that participated in the study. The data underlying this article will be shared on reasonable request to the corresponding author.
